# Thermal, Electrical and Surface Hydrophobic Properties of Electrospun Polyacrylonitrile Nanofibers for Structural Health Monitoring

**DOI:** 10.3390/ma8105356

**Published:** 2015-10-14

**Authors:** Ibrahim M. Alarifi, Abdulaziz Alharbi, Waseem S. Khan, Andrew Swindle, Ramazan Asmatulu

**Affiliations:** 1 Department of Mechanical Engineering, Wichita State University, 1845 Fairmount, Wichita, KS 67260-0133, USA; imalarifi@wichita.edu (I.M.A.); alharbi.wsu@gmail.com (A.A.); 2 Department of Mechanical and Industrial Engineering, Majmaah University, Riydh, Saudi Arabia; waseem_beacon@yahoo.com; 3 Department of Geology, Wichita State University, 1845 Fairmount, Wichita, KS 67260-0133, USA; Andrew.Swindle@wichita.edu

**Keywords:** electrospinning, polyacrylonitrile (PAN) nanofibers, oxidation, carbonization, physical properties

## Abstract

This paper presents an idea of using carbonized electrospun Polyacrylonitrile (PAN) fibers as a sensor material in a structural health monitoring (SHM) system. The electrospun PAN fibers are lightweight, less costly and do not interfere with the functioning of infrastructure. This study deals with the fabrication of PAN-based nanofibers via electrospinning followed by stabilization and carbonization in order to remove all non-carbonaceous material and ensure pure carbon fibers as the resulting material. Electrochemical impedance spectroscopy was used to determine the ionic conductivity of PAN fibers. The X-ray diffraction study showed that the repeated peaks near 42° on the activated nanofiber film were α and β phases, respectively, with crystalline forms. Contact angle, thermogravimetric analysis (TGA), differential scanning calorimetry (DSC) and Fourier transform infrared spectroscopy (FTIR) were also employed to examine the surface, thermal and chemical properties of the carbonized electrospun PAN fibers. The test results indicated that the carbonized PAN nanofibers have superior physical properties, which may be useful for structural health monitoring (SHM) applications in different industries.

## 1. Introduction

### 1.1. General Background

The process of enacting an organized damage detection system for various types of infrastructure is generally referred to as structural health monitoring (SHM) [1]. SHM provides continuous monitoring of structures in real time, thereby increasing public safety, especially for aging structures still in use [2,3,4,5]. SHM ensures early detection of routine damage and reduces the costs and down-time associated with maintenance. SHM can predict the unusual behavior of various structures, providing advanced warning and allowing for the removal/repair of structural parts for the protection of the public [5]. In addition to monitoring any unsafe structural behavior, SHM also allows for the assessment of structural integrity after natural disasters, such as earthquakes, floods, tornados and hurricanes. The need for SHM is obviously important, with the primary objectives of such a system being to increase safety and reliability further and to reduce repair and inspection costs [5].

Carbon nanofibers possess high stiffness and strength with outstanding electrical and thermal properties; therefore, they have numerous applications in a wide range of fields, such as structural components, electronics, medical, filtration and lithium-ion batteries [6,7,8,9,10]. Carbon fibers are also being used as reinforcement in manufacturing lightweight composites for aircraft and wind turbines. Modern PAN-derived carbon fibers were first developed by Shindo after pyrolyzing polyacrylonitrile (PAN) fibers at elevated temperatures in an inert atmosphere. The development of commercial carbon fibers was reported by Watt *et al.* [11] after Shindo’s discovery.

The main precursor for the production of carbon fibers is polyacrylonitrile (PAN). Electrospinning, which involves using an electric current to draw a liquid into a thin fiber, is a promising technique for the production of carbon nanofibers from a PAN polymer precursor [6,7]. PAN-derived carbon nanofibers were synthesized via electrospinning followed by stabilization/oxidation and carbonization to remove all non-carbonaceous matter and to ensure a high percentage of carbon in the resulting material. The stabilization process of PAN fibers is extremely important in order to fabricate high-quality carbon fibers. In this process, a lower temperature treatment (200–300 °C) in ambient conditions results in the formation of the ladder-like structure of PAN [11]. During the stabilization process, PAN precursor fibers undergo significant physical and chemical changes and become physically, chemically and thermally stable when exposed to a high temperature [12]. The PAN fibers are transformed from thermoplastic to thermosetting and change color as well during the heating process. Additionally, PAN fibers are shrunk during stabilization processes, resulting in weight loss and fiber diameter reductions. The critical parameters in stabilization are temperature, heating steps, heating rate, inert atmosphere and holding time.

After the stabilization process, carbonization is carried out at a higher temperature (800–3000 °C) in an inert atmosphere [11]. The carbonization temperature depends on the types of carbon fibers required. The properties of the carbon fibers depend on the nature of precursor fibers and the process parameters used in stabilization and carbonization [11]. In the present study, electrospun PAN nanofibers were converted into carbon fibers by stabilizing and carbonizing at high temperatures, which results in high strength materials due to the higher carbon contents. PAN contains around 68 wt % carbon, and after heat treatment, the carbon content escalates to around 92 wt %. The aim of this study was to fabricate electrospun PAN nanofibers (PAN-derived carbon fibers), to heat treat them to remove all non-carbonaceous material and to investigate their thermal, electrical, surface and structural properties for potential applications in SHM systems.

### 1.2. Polyacrylonitrile Electrospun Fibers as a Strain Sensor in Structural Health Monitoring Applications

SHM has gained tremendous attention recently as a means to accurately monitor structures and proffer an early warning of a detrimental condition using real-time response data. By using structurally-integrated sensors, the health of a structure can be interpreted by means of sensor signals, and real-time data inspection can reduce the extra cost of inspection. Strain is one of the most important parameters to investigate the health of a structure [13]. Repetitive strains with high magnitudes may lead to fatigue or yielding in the material. Mechanical strains can be used to determine structural loads and stresses in the structure [13]. Conventional strain sensors are generally made from metal foils and possess several drawbacks, such as limited sensitivity, early failure, high-temperature dependence and large power consumption [13]. Thus, they are deficient for low power consumption and high performance [13,14,15]. An efficient sensor for SHM has great potential for applications in monitoring aerospace structures subjected to fatigue, impact, severe loads and structural disintegration. Strain sensing is generally attained by attaching strain gauges to the structures [16].

Sihai *et al.* [16] used carbon fibers in a cement structure and studied the sensing performance of carbon fibers. Recently, many studies have been done with carbon nanotubes embedded in a polymer matrix to investigate sensing performance. Among various carbon fiber precursors, Polyacrylonitrile (PAN) has been used extensively for producing carbon fibers for the last several decades, owing to its large carbon yield. With the advent of the electrospinning technique of producing fibers, ultrafine nanosized fiber webs have been produced recently, having a much smaller diameter in comparison to conventional PAN-based carbon fibers and a higher specific surface area. Carbon fibers are electrically conductive, and when subjected to loads or strains, they experience a change in electrical resistance in response to strain, thus enabling strain sensing ability [17]. The electrical conduction of carbon fibers is due to its graphite microstructure of high order [18]. Loadings or strains, therefore, induce a shift in electrical resistance within the fibers, accordingly enabling them to be used as a sensor. Moreover, carbon fibers display piezoresistive properties. Mäder *et al*. [18] studied the strain sensing performance of single carbon fibers coated with a conductive material for SHM applications. The strain sensing performance of carbon fiber composites depends on several factors, such as fiber volume fraction, continuous fibers, discontinuous fibers, randomly-oriented fibers, short fibers and unidirectional fibers. When highly thermal and electrically-conductive carbon fibers are used in composites with a higher volume fraction, the carbon fiber-reinforced plastics (CFRP) becomes somewhat conductive. Epoxy is an insulator, whereas carbonized PAN-derived carbon fibers are conductive. The electrical SHM depends on the material behavior of the sensors. When microcracks and/or delamination occur during the service of a composite structure, the electrical conduction is impeded, as the damaged area restricts the flow of electrons. When carbonized PAN-derived carbon fibers are used in the composite structure, they can detect microcracks and/or delamination and serve as sensors.

## 2. Results and Discussion

### 2.1. X-ray Diffraction *(*XRD*)* Analysis

The X-ray diffraction (XRD) technique was used for the structural characterization of carbon materials to provide useful information regarding lattice constants and diffraction planes [19]. Cu Kα (λ = 0.15418 nm) radiation was used over the 2θ range of 10°–70° to observe the structural change during the carbonization. An X-ray beam directed at a right angle to the samples produced reflections from the planes. The apparent crystallite thickness (*L*c), the apparent layer-plane length parallel to the fiber axis (*L*a) and the average interlayer spacing *d* were calculated using Bragg’s and Scherrer’s formulas. Bragg’s and Scherrer’s formulas can be expressed, respectively, as [20]:

(1) $$d=\frac{\text{λ}}{2\;sin\text{θ}}$$

(2) $$L=\frac{K\text{λ}}{\text{β}cos\text{θ}}$$

where θ is the Bragg angle in degrees, λ is the wavelength of the X-ray used and β is the full-width half maximum of a given peak (rad). The shape factor K is 0.89 for *L*c and 1.84 for *L*a, respectively [20]. Scherrer’s equation was used to calculate the values of *Lc* (stacking height of layer planes) from the width of the (002) reflection.

**Figure 1:**
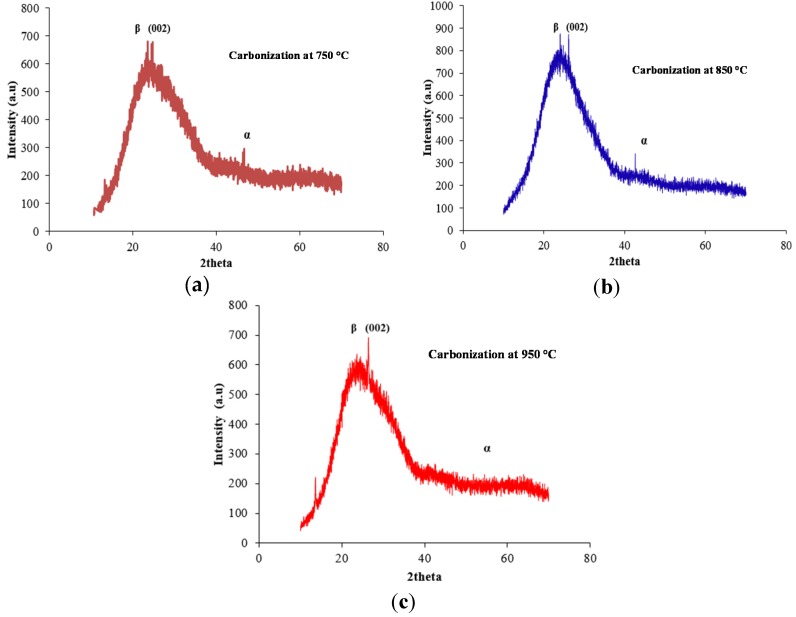
(**a**) X-ray diffraction (XRD) pattern of PAN pre-carbonized fibers at 750 °C; (**b**) XRD pattern of PAN pre-carbonized fibers at 850 °C; (**c**) XRD pattern of PAN pre-carbonized fibers at 950 °C.

**Figure 2:**
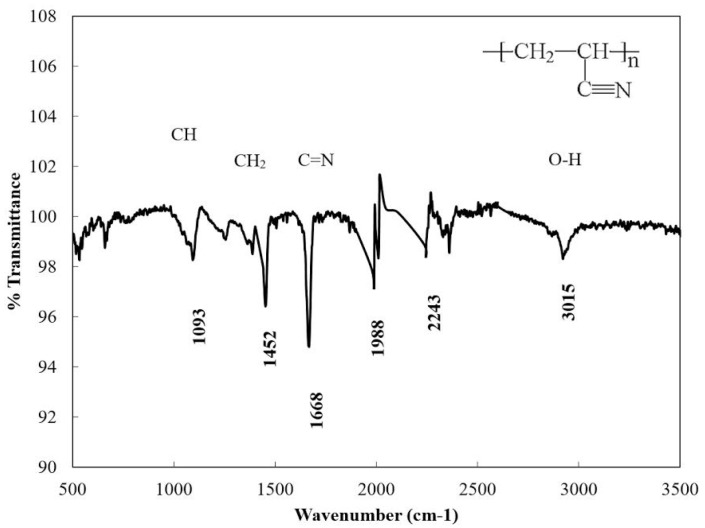
The Fourier Transform Infrared Spectroscopy (FTIR) Spectra of the Polyacrylonitrile fibers prior to carbonization.

**Figure 3:**
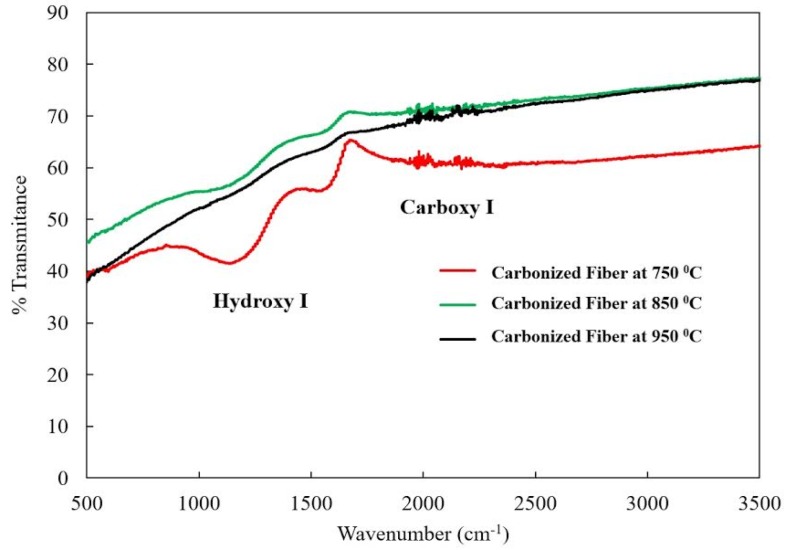
The Fourier transform infrared spectroscopy (FTIR) spectrum of carbonized (PAN) fibers at 750, 850 and 950 °C.

Figure 1 shows the XRD patterns along the (002) plane of the PAN pre-carbonized fibers at various temperatures (750, 850 and 950 °C). As can be seen in Figure 2 and Figure 3, when the carbonization temperature increases, the (002) peaks shift from higher values of Bragg angles to slightly lower values of Bragg angles. These results show that carbon atoms arrange themselves into an increasingly ordered form as the carbonization temperature increases, resulting in improvement of the crystalline structure of the fibers [20]. According to Bragg’s law, as 2θ decreases, the corresponding d-spacing increases (*i.e*., the lower angles mean a larger d-spacing). Table 1 gives the structural parameters of the XRD for the carbonized PAN fibers.

**Table 1:** The structure parameters determined by X-ray diffraction for the PAN carbon fibers.

Temperature (°C)	*d*_(002)_ nm	*L*_c_ nm	*L*_c_/*d*_(002)_
750	0.4053	2.66	1.87
850	0.4128	2.76	1.58
950	0.4226	2.76	1.58

### 2.2. Fourier Transform Infrared Spectroscopy *(*FTIR*)* Analysis

FTIR spectra are considered to be a useful tool for determining the chemical interaction during the heat treatment of the PAN fibers. With the help of the spectra, it is possible to study the relaxation between chemical changes and strength [21]. As is seen in Figure 2, the vibration characteristic of the PAN fibers are those of the nitrile group corresponding to 1668 cm^−1^, and the peak intensities at 2922, 1452, 1093 cm^−1^ are assigned to the aliphatic CH group vibration of different modes in OH, CH_2_ and CH, respectively. The band corresponding to 1988 cm^−1^ is due to C=O stretching, while the peak corresponding to 1668 cm^−1^ is due to nitrile stretching, indicating the presence of acrylonitrile.

The PAN molecule consists of methyl (CH_3_) and nitrile (C≡N) groups in a linear arrangement [22]. During the oxidation step, some new compounds, such as ketones, aldehydes and carboxylic acid, are formed. The carbonization process results in the evolution of the volatile compounds, leaving behind primarily carbon and hydrogen molecules in the carbon fiber structure. However, 100% conversion of PAN polymer to carbon may not be achieved due to the existence of other compounds [22]. There are several distinct changes in the characteristic absorption peaks at some locations of the FTIR spectrum following carbonization [23]. Significantly, the strongest and sharpest peaks disappear after carbonization (Figure 2 and Figure 3). The strong peak from the nitrile groups observed at 1668 cm^−1^ (Figure 3) has almost disappeared after carbonization, especially at 850 and 950 °C. The peak at 1668 cm^−1^ decreased due to the reaction of the nitrile group during the cyclization to form the conjugate C=N group [23]. No peak at 1668 cm^−1^ was observed in the 850 °C curve, indicating that nitrile groups no longer exist and no ladder-like structure forms after carbonization at 850 °C. A similar behavior was observed in the 950 °C spectrum; however, several very small peaks are visible, which could be due to the sensitivity of the device or noise. The peak associated with the hydroxyl group at 2922 cm^−1^ completely disappeared after carbonization. The peak corresponding to 1452 cm^−1^ shows the bending vibration from the CH_2_ group (Figure 2). FTIR spectra following carbonization at 750 °C still exhibited distinct peaks corresponding to 1093, 1452 and 1668 cm^−1^ due to CH, CH_2_ and C=N group formations, respectively. However, as the carbonization increased to 850 and 950 °C, the distinct peaks disappeared in FTIR spectra. The disappearance of these peaks can be attributed to the evolution of gasses and some liquid compounds and conversion into carbonaceous materials.

As can be seen in Figure 3 (carbonization at 750 °C), the presence of peaks in the FTIR spectra between 1093 and 1700 cm^−1^ indicate that 750 °C was insufficient to fully carbonized the PAN fibers or that some reactions might not be complete. The lack of peaks over this range in the 850 °C curve reveals that the PAN fibers have been almost fully carbonized. The reappearance of small peaks in the 950 °C curve is most likely due to the experimental conditions or the sensitivity of the equipment/noise. The magnitude of the peaks is still consistent with the PAN fibers being nearly fully carbonized at this condition or the sensitivity of the equipment/noise. The small wriggling seen in the 950 and 750 °C curves may be attributed to the minute variations in thickness in a different sample. The sample thickness was between 1.12 and 1.14 mm. PAN has the tendency of absorbing moisture from the environment, and it can absorb water vapor, as well. This could be the possible reasons for wriggling behavior in the 950 and 750 °C curves. The heating rate was 5 °C/min in all experiments.

### 2.3. Thermogravimetry Analysis *(*TGA*)* Analysis

**Figure 4:**
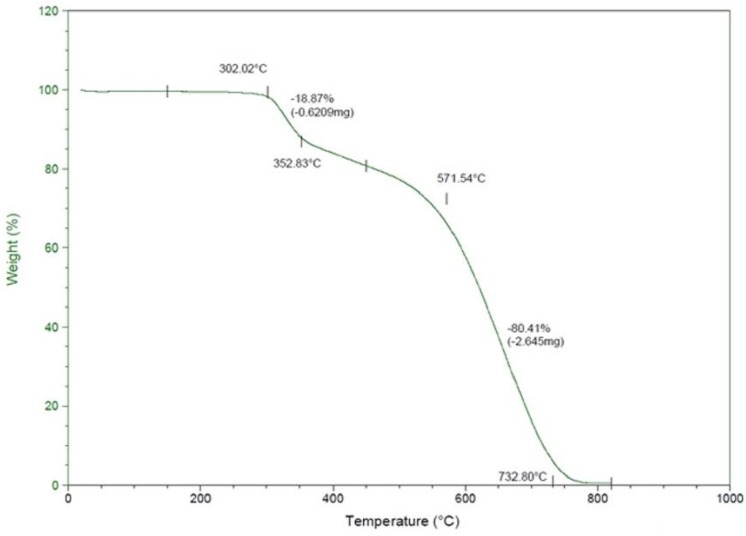
The Thermogravimetry analysis (TGA) curve of the PAN fibers prior to the carbonization.

Thermogravimetry analysis (TGA) offers a quantitative analysis of the amount of moisture and volatile compounds present in the fibers, weight loss and thermal breakdown and also assists in determining the degradation mechanism. It is important to note that an accurate heating rate is critical for thermogravimetry. TGA was employed to determine the weight loss pattern of the PAN fibers as a function of temperature. TGA analysis was conducted on fiber samples from 25–850 °C with a nitrogen flow rate of 60 mL/min and a heating rate of 10 °C/min. The samples used for the TGA tests contained 3.237–5.5660 mg of fibers. The measurements were started at room temperature (25 °C) and ramped to 850 °C at a heating rate of 10 °C/min using a nitrogen purge rate of 60 mL/min in a platinum pan. Figure 4 show the thermogravimetric curves of PAN fibers.

The heating of the PAN fibers induces chemical reactions, such as cyclization, degradation and thermal cross-linking, and the mechanisms of these processes depend on the heating rate, the medium, the mass of the polymer and the type and nature of filler materials [24]. The TGA curve of PAN fibers shows four-step weight loss patterns. The TGA curves demonstrate that the weight loss caused by the pyrolytic reactions mainly occurred at around 302 °C for pure PAN. The cyclization reactions continues until the residual nitrile groups do not react with other functional groups, and the pyrolytic reactions of the PAN fibers proceed with minimum heating and shrinkage effects [25]. The cyclization of the fibers proceeds before any mass loss, and the factor responsible for the cyclization process is the formation of aromatic rings [26]. In the first stage up to 302.02 °C, there was no weight loss. The cyclization reaction takes place in this step, and cyclization does not cause any weight loss, theoretically [27].

**Figure 5:**
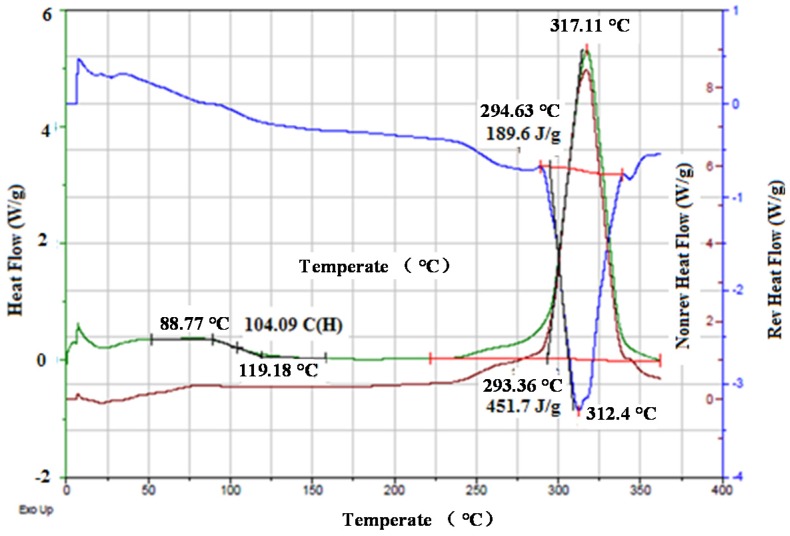
The DSC thermogram of the PAN fibers for heat flow, non-reverse heat flow and reverse heat flow.

In the second stage (between 302.02 and 352.83 °C), the weight loss is 18.87%, indicating that a chemical reaction occurred and that volatile gasses were gradually released. It should be noted that the cyclization peak temperature is 294.63 °C from the DSC thermogram (Figure 5). In the third stage, some weight loss was also observed between temperatures of 382 and 571 °C, which could be attributed to partial evaporation of NH_3_ and HCN [27]. In the fourth stage, between temperatures of 571 and 732 °C, a steady decrease in weight was observed, with a total loss of 80.41%, indicating complete evaporation of polymer chain fragments from the PAN fibers. The TGA analysis of PAN fibers has shown that the degradation process of the PAN fibers is exothermic and is accompanied by the evolution of the volatile gaseous products.

### 2.4. Differential Scanning Calorimeter *(*DSC*)* Analysis

**Figure 6:**
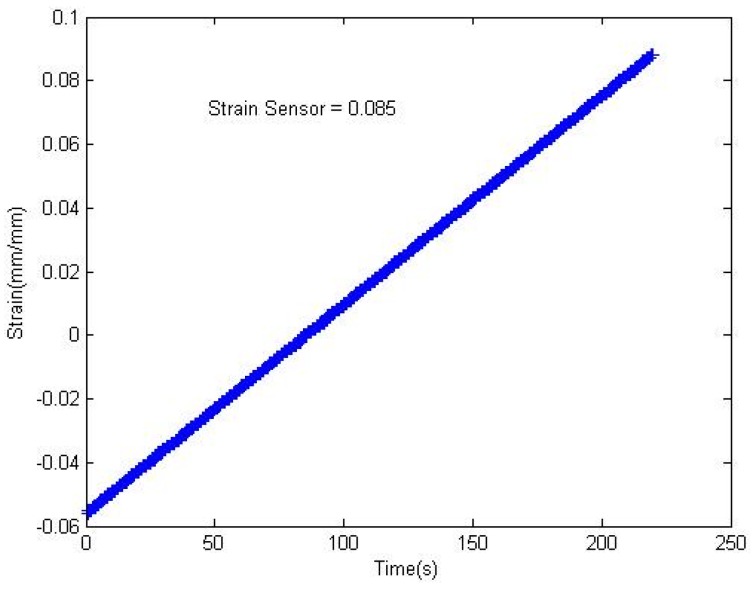
Strain *vs.* time curve.

A DSC Q1000 (TA instrument, New Castle, USA) interfaced with a PC was used to measure the thermal properties of the PAN fibers. Glass transition temperature (T_g_) and melting temperature (T_m_) were investigated with a heating rate of 20 °C/min and a nitrogen flow rate of 50 mL/min. The samples were sealed in a T_zero_ PAN (TA Instruments), and the measurements were conducted in the temperature range of 25–350 °C. A pre-determined weight of each sample was used in this experiment. The DSC heat flow and temperature were calibrated with an iridium standard prior to the tests. Figure 6 reveals the DSC thermogram of the PAN fibers.

As is seen in Figure 5, the glass transition temperature of the PAN fibers is 104 °C. No melting was observed with DSC, as indicated by the green line. However, melting was observed with modulated DSC (modulated 1.5 °C/60 s), as indicated by the blue line. The PAN fibers show a broad exothermal peak at 312.4 °C (Figure 6). The onset temperature was 294.63 °C, and the reaction was completed at 330 °C. The broadening of the exothermic peak may be due to the cyclization process. The broadening of the exothermic peak and corresponding temperature depends on the stabilization time. As the stabilization time increased, the cyclization peak broadened, and the cyclization temperature considerably increased. The PAN fibers are cyclized only by a free radical mechanism, thereby indicating one peak at 312.4 °C. The cyclization of nitrile groups is highly exothermic and leads to the fragmentation of the chains owing to the heat building up rapidly in the sample [28]. The single exothermic peak observed in the DSC thermograms can be attributed to the cyclization of the PAN molecular chains [25]. It is well known that the cyclization reactions in PAN are based on the free radicals and initiated high temperature [29]. Therefore, the single exothermic peak observed in PAN can be attributed to the overlapping of the free radical cyclization and other exothermic reactions that can take place at high temperatures [29]. Cyclization is an extremely exothermic process, and it is accompanied by the evolution of a large amount of heat flow that can cause the cyclization of the nitrile group in PAN [29]. In the TGA thermogram (Figure 5), the cyclization continued without any weight loss until the temperature reached 302.02 °C; nevertheless, the cyclization peak in the DSC thermogram (Figure 6) was observed at 312.4 °C.

### 2.5. Ionic Conductivity Test

A GAMRY Instrument (Reference 600, Potentiostat/Galvanostat/ ZRA, Warminster, PA, USA) was employed to characterize the ionic conductivity of the PAN nanofibers. From Equation (3), the ionic conductivity can be calculated at different temperatures. The conductivity values of the polymeric fibers were calculated using the resistance obtained from a slope of the I–V plot (I: Unit of current, V: unit of voltage).

(3) $$\text{σ}=\;\frac{L}{R\;A}$$

where “*A*” is the area of the film and “*L*” is the thickness of the film. The addition of conducting moieties in the polymer matrix can also increase the ionic conductivity [30,31,32]. Table 2 shows the conductivity values of the carbonized PAN fibers at 850 °C at different temperatures.

**Table 2:** The conductivity values of carbonized PAN fibers at 850 °C.

Temperature (°C)	Conductivity (σ) S/cm (×10^3^)
24.85	2.13
25.17	1.85
35.10	2.78
39.13	3.31
40.10	4.31
42.01	4.50
43.88	4.48
57.60	4.49
77.40	4.51

Generally, the conductivity increases with increasing temperature [33]. Changing carbonization environments does not have much influence on the electrical and ionic conductivity values of the samples; however, the carbonization temperature is the only parameter that plays a major role in conductivity values [33]. The carbonized PAN nanofibers (inorganic phase) have much higher electrical conductivity than that of polymer nanofibers (organic phase) without any carbonization [34]. Carbonization at a high temperature generally increases the conductivity due to the reduction of sp^3^ bonds [34]. No significant changes in conductivity were observed between parallel and perpendicular directions. The electrical conductivity of most polymers is in the order of μs/cm. The electrical conductivity of an individual PAN nanofiber after carbonization is 4.9 s/cm [35]. However, the conductivity of PAN-derived carbon fibers depends on the carbonization temperature on the carbon content of PAN-derived carbon fibers. Generally, the PAN-derived carbon fibers contain around 92 wt % carbon; however, when they are carbonized at 2000–3000 °C, they would contain 99 wt % carbon. As is seen in Table 2, the conductivity values increase with increasing temperature. The highest value of conductivity is at 350.55 °C, whereas the lowest value of conductivity is at 298 °C. The carbonization was carried out at 850 °C in this study, which means that the PAN fibers may still contain a small amount of impurities, and these impurities may reduce the conductivity values to some extent. If the carbonization is carried out at around 3000 °C, the fibers will have a pure graphite structure with nearly 99 wt % carbon, thereby showing the highest values of electrical conductivity.

### 2.6. Surface Hydrophobicity of Carbonized Fibers

The hydrophilic and hydrophobic properties of polymer surfaces are determined by the water contact angle between the water droplet and the polymeric fiber surface. A hydrophobic surface is one on which a droplet of water forms a contact angle greater than 90°, whereas a hydrophilic surface is one on which a droplet of water forms a contact angle less than 90° [36]. Polymer surfaces with the contact angle between 150° and 180° are called superhydrophobic [37]. This phenomenon is also known as the “lotus effect”, which exhibits self-cleaning and anti-contamination features [13,37,38]. Table 3 shows the water contact angle values of the PAN fibers at different carbonization temperatures.

**Table 3:** The contact angle values for electrospun carbonized PAN fibers.

No.	Water Contact Angle (°)					
	750 °C		850 °C		950 °C	
	Right	Left	Right	Left	Right	Left
1	149.46	152.36	156.22	156.32	149.21	154.78
2	151.32	154.85	155.23	157.22	152.55	153.22
3	152.63	149.98	155.69	153.45	156.33	154.32
4	153.55	151.11	159.63	155.25	157.54	156.33
5	159.90	148.79	159.58	156.08	158.64	152.52
Mean	152.40		156.47		154.54	
Standard Deviation (SD)	±3.23		±1.92		±2.80	

The water contact angle on solid surfaces depends on surface chemistry and surface morphology. PAN fibers are semi-crystalline, with some degree of polarity due to radicals in the chain structure, which may affect the water contact angle values. Prior to the carbonization, the PAN nanofibers provided a water contact angle of about 95°, which may be because of the moisture absorbed, the porosity and the presence of beads left from the electrospinning process. Due to these material defects, PAN has the tendency to absorb water under normal conditions and also to absorb some moisture from the atmosphere, as well. However, during the carbonization process, the surface chemistry and surface morphology of the PAN fibers are significantly altered. Specifically, the pores and beads in the material are diminished, and the polarity due to the radicals attached to the main chain is eliminated as non-carbonaceous compounds are released. The carbonized PAN fibers fall into the superhydrophobic material range, with water contact angles between 152° and 156°. The carbonized PAN fibers at 750 °C had an average water contact angle of 152.40° (±3.23°), while the fiber carbonized at 850 °C provided the highest average water contact angle (156° ± 1.92°). Interestingly, the average water contact angle decreased slightly (154° ± 1.54°) when the carbonization temperature was increased to 950 °C, although it is still within the superhydrophobic range. As can be seen in Table 3, the carbonized PAN fibers at 850 °C show the highest value of the water contact angle. This could be attributed to the absence of any residual element in the fibers.

The FTIR results (Figure 5) for 850 °C confirm that there is no wriggling in this curve. However, the other two curves for 750 and 950 °C show some spikes in FTIR spectra. Likewise, the contact angle values for these two samples (750 and 950 °C) display a declining trend due to the presence of some residual elements in the fiber texture. PAN can absorb moisture and suspend water vapors from the atmosphere, which may lead to heterogeneity in the samples and cause small variations in contact angle values and some spikes/wriggling in the FRIT spectra. Apart from these minor differences in contact angle values, all of the samples are sill superhydrophobic. The superhydrophobicity of these PAN fibers may be useful in fabricating pre-preg nanocomposites. These pre-preg nanocomposites with PAN-derived carbon fibers placed at the top of the composite plies having superhydrophobic characteristics can be attached with composite structures and serve as strain sensors due to their excellent electrical conduction and piezoresistive characteristics.

### 2.7. Strain Sensing Performance

**Figure 7:**
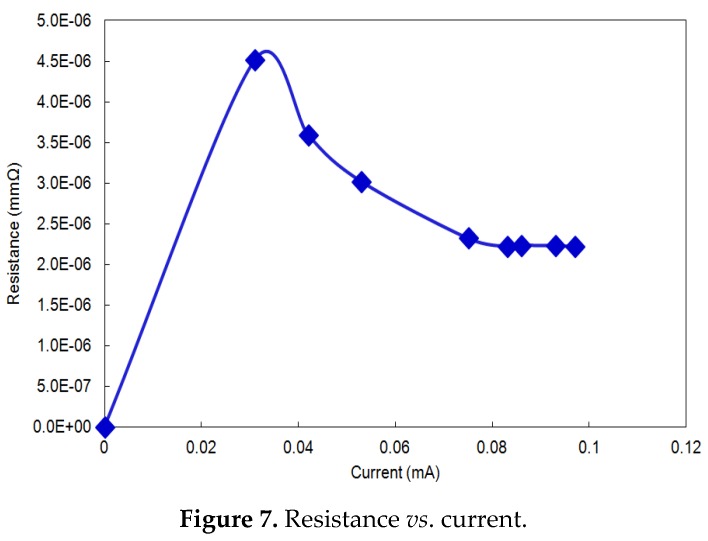
Resistance *vs*. current.

Figure 6 and Figure 7 show the strain *vs.* time and current *vs.* resistance curve, respectively, for the nanocomposite specimen used in this study. The nanocomposite contained pre-preg carbon plies and carbonized PAN fiber at the top of the composite assembly, which means that the composite is highly conductive. Figure 7 shows the resistance of the nanocomposite specimen against the current when the tensile loadings are applied. The change in strain (Figure 6) can be observed as the specimen undergoes cyclic loadings, and a corresponding change in resistance can be observed, as shown in Figure 7. The cyclic loadings may induce microcracks inside the nanocomposite specimen, which can be observed as a change in resistance values. Strains induce a change in resistance within the fibers’ texture, impeding the flow of free electrons. Figure 7 shows the corresponding change in the electrical resistance. The size and orientation of PAN-carbonized fibers has a significant impact on the current distribution and change in resistance. A similar study done by Thostenson and Chou reported a maximum percentage change in resistance of around 300% due to damage in glass fiber-epoxy composites at a peak load [39]. According to another study, a maximum percentage resistance change of about 40% due to damage in CNT-reinforced laminated bidirectional woven glass fiber epoxy composites was reported during tensile and fatigue loading [39].

As is seen in Figure 7, the resistance increases rapidly at the beginning, as the current increases, which corresponds to the loadings. As soon as the maximum stress is reached, the resistance decreases due to the relaxation of the material between breaking and maximum stress.

## 3. Experimental

### 3.1. Materials

PAN (molecular weight 150,000 g/mole (CAS No. 25014-41-9, St. Louis, MO, USA) and dimethylformamide (DMF) (CAS No. 68-12-2, 99.8%, St. Louis, MO, USA) were purchased from Sigma-Aldrich (St. Louis, MO, USA) and used without any further purification. These chemicals were used in the tests without any modifications.

### 3.2. Method

#### Carbonization of Polyacrylonitrile (PAN) Nanofibers

**Figure 8:**
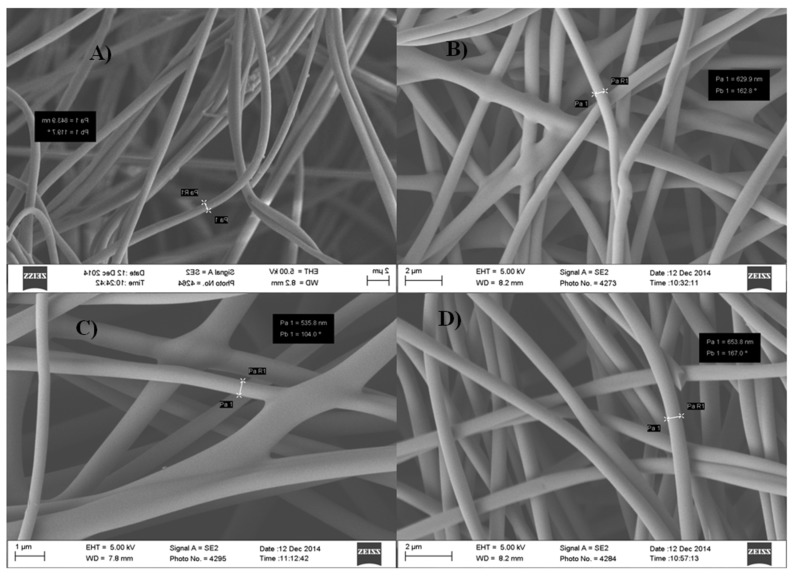
SEM images of PAN-derived carbon fibers: (**a**) the electrospun PAN fibers; (**b**) the same fibers after the carbonization at 750 °C for 1 h; (**c**) 850 °C for 1 h; and (**d**) at 950 °C for 1 h.

PAN powder was dissolved in DMF with a 90:10 weight ratio at 500 rpm for 1 h. Special care was taken to ensure a homogeneous blend (polymeric solution). Nanofibers were fabricated using electrospinning at 25 kV with a 1 mL/h feed rate and a tip-to-collector distance of 25 cm. The as-produced nanofibers were later converted into carbon nanofibers by first stabilizing (or oxidized) in an oxygen atmosphere at 270 °C for 1 h. Then, the carbonization of PAN fibers was performed by heating the stabilized PAN fibers at 750, 850 and 950 °C in an inert atmosphere (argon atmosphere) for 1 h. The heating ramp rate was 5 °C/min. After the heat treatment, the non-carbon elements, such as hydrogen, oxygen, nitrogen or sulfur, were eliminated and released as volatile matter, leaving behind high carbon content nanofibers [12,23,40,41,42,43]. Prior to the electrical and thermal tests, the carbonized nanofibers were treated with hydrochloric acid (HCl) in order to activate the carbon fibers and to enhance the electrical and thermal conductivities. The acid solution was prepared by mixing 3.5 g of HCl into 100 mL of Deionized (DI) water. Scanning electron microscopy (SEM) was used to determine the surface morphology of the PAN-derived carbon fibers. Figure 8 shows SEM images of the PAN fibers before and after carbonization at different temperatures.

A Rigaku MiniFlex II XRD system (Rigaku America Corporation, The Woodlands, TX, USA) was used for the structural characterization tests. Fourier transform infrared spectroscopy (FTIR) analysis was done by using a Thermo Scientific™ Nicolet™ iN10 Infrared Microscope (Plam Beach, FL, USA) in the range of 500–3500 cm^−1^ with an attenuated total reflectance (ATR) mode. The thermal stability of PAN fibers was studied by means of thermogravimetric analysis (TGA) using a Q500 TA instrument (New Castle, USA). A differential scanning calorimetry (DSC) Q1000 (TA instrument) interfaced with a PC was used to measure the thermal properties of PAN fibers. A GAMRY Instrument (Reference 600, Potentiostat/Galvanostat, ZRA, Warminster, PA, USA) was employed to measure the ionic conductivity of PAN samples. The tensile test was done by a Tensile Test MTS Criterion Model 44 Advantage™ High Elongation Extensometer (Eden Preairie, MN, USA). The contact angle values of PAN fibers were measured by a water contact angle goniometer (KSV Instruments Ltd with Model# CAM 100, Monroe, LA, USA). The contact angle goniometer is a compact video-based instrument, which measures contact angles between 1° and 180° with an accuracy of ±1°. Computer software provided by KSV Instruments Ltd (Monroe, LA, USA) precisely records and measures the contact angles and also takes pictures of the measured contact angle.

### 3.3. Fabrication of Nanocomposite for Sensors

**Figure 9:**
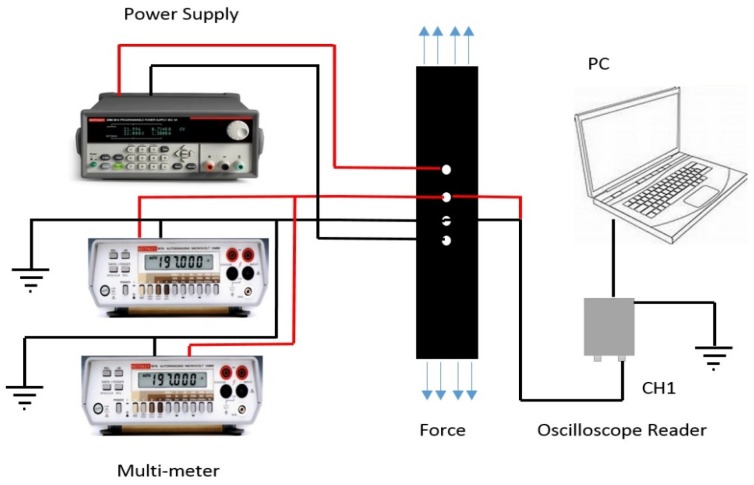
Experimental set up for the SHM strain sensor.

The strain-sensing performance of PAN-derived carbon fibers was tested by fabricating a nanocomposite employing the pre-preg technique. The PAN carbonized fibers was placed at the top, and pre-preg carbon plies were laid up following a 0, 45, −45 and 90 stacking sequence on an aluminum mold. The mold was placed in an oven for 2 h at 180 °C for curing. A four-circumferential ring probe was used to measure the resistance of the samples. A constant current having small magnitude was applied along the axial direction through the outer probes, and variation in voltage between inner probes was recorded, corresponding to the change in strain, as is shown in Figure 9. Quasi-static loading was applied, and the specimen was loaded at a constant rate of 1 mm/min.

## 4. Conclusions

PAN is one of the most extensively-studied polymers with a wide range of industrial applications (e.g., aircraft, wind turbine, automobile, *etc.*). In this study, PAN-based electrospun fibers were oxidized at 270 °C and subsequently carbonized at 750, 850 and 950 °C for 1 h to fabricate highly purified carbon nanofiber mats. This study deals with the property analysis of the carbonized electrospun PAN fibers for possible SHM system development. Several characterization techniques, such as EIS, XRD, TGA, DSC, FTIR and water contact angle measurements, were conducted on the PAN fibers before and after the carbonization processes in order to investigate the chemical, electrical, structural and surface properties of the PAN-based fibers. The test results indicate that after the carbonization of the PAN nanofibers’ physical properties, such as carbon wt %, hydrophobicity and ionic conductivity, the nanofibers were drastically improved, which may be useful for the various industries that employ SHM systems.
